# Urticaire fessier géant en consultation dermatologique chez une secrétaire de bureau

**DOI:** 10.11604/pamj.2021.38.390.29225

**Published:** 2021-04-21

**Authors:** Yamoussa Karabinta, Mamadou Gassama

**Affiliations:** 1 Centre Hospitalier Universitaire de Dermatologie de Bamako, Bamako, Mali,; 2 Faculté de Médecine et d´Odontostomatologie de Bamako, Bamako, Mali

**Keywords:** Urticaire chronique, géant, secrétaire de bureau, Chronic urticaria, giant, office secretary

## Abstract

Urticaria is a fleeting papulous edematous pruritic rash on the skin, most often caused by an allergic reaction (food, drugs, insect bites, etc.). It can be acute, lasting less than 6 weeks or chronic, with recurrent episodes for more than 6 weeks. Chronic urticaria (UC) is a frequent reason for consultation. Patients management is often difficult due to its significant impact on the quality of life, its potential association with many underlying diseases and a sometimes insufficient response to first line treatment. The prevalence of urticaria in the general population is 0.6%-1.3%. Women are more frequently affected than men (3.8 times). Therapy is focused on reducing pruritus, size, number and frequency of lesions and is based on antihistamines and avoidance of exposure to contributing factors. We here report a case of chronic giant urticaria on the gluteal region in a 36-year-old office secretary with a history of chronic urticaria treated with Loratadine (10 mg tablet, once daily), presenting with edematous pruritic papules on the buttocks occurred more than 3 days before. Physical examination showed large erythematous edematous pruritic papules coalescing into large plaques on both buttocks. The largest plaques measured 25/20 cm, while the smallest 4 cm/3cm. Complete blood count was performed, which showed leukocytosis (mainly eosinophilic polynuclear leukocytosis). The diagnosis of urticaria was retained based on clinical lesions. The patient received 40mg Solumedrol for injection (2 ampoules for 5 days) and Bilastine (20 mg tablet per day). Outcome was favorable under treatment, with complete remission of lesions and disappearance of pruritus.

## Image en médecine

L´urticaire correspond à une éruption papuleuse œdémateuse prurigineuse fugace sur la peau dont la cause est le plus souvent allergique (aliments, médicaments, piqûres d'insectes…). Il peut être aiguë d´une durée inférieure à 6 semaines ou chronique se répétant sur plus de 6 semaines consécutives. L´urticaire chronique (UC) est une cause fréquente de consultation. Sa prise en charge est souvent difficile en raison de son impact important sur la qualité de vie, de son association potentielle avec de nombreuses pathologies sous-jacentes et d´une réponse parfois insuffisante au traitement de première ligne. Sa prévalence est estimée à 0,6%-1,3% de la population générale. Les femmes sont plus fréquemment touchées que les hommes (3,8 fois). Son traitement consiste à une diminution du prurit, de la taille, du nombre et de la fréquence des lésions par les antihistaminiques et l´éviction des facteurs favorisants. Nous rapportons un cas d´urticaire chronique fessier géant chez une secrétaire de bureau âgée de 36 ans aux antécédents d´urticaire chronique traitée par Loratadine 10mg en raison d´un comprimé par jour qui présente depuis plus de 3 jours des papules œdémateuses prurigineuses sur les fesses. A l´examen physique, nous avons constaté de grosses papules érythémateuses, œdémateuses prurigineuses confluent en gros placards dont le plus gros mesure 25cm/20cm et le plus petit 4cm/3cm localisées sur les deux fesses. Une numération formule sanguine réalisée a objectivé une hyperleucocytose à prédominance polynucléaire éosinophile. Le diagnostic de l´urticaire a été retenu devant les lésions cliniques. La patiente a reçu comme traitement, le Solumedrol 40mg injectable en raison de 2 ampoules par jour pendant 5 jours et le Bilastine 20mg comprimé, 1 comprimé par jour. L´évolution sous ce traitement était favorable avec rémission complète des lésions et disparition du prurit.

**Figure 1:**
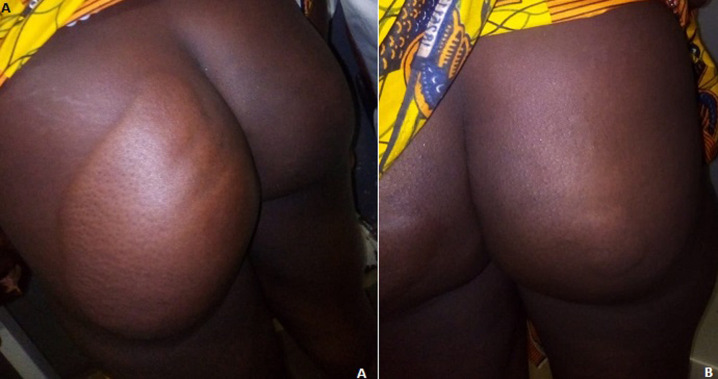
A) papules œdémateuses prurigineuses confluent en placard; B) multiples papules œdémateuses sur les fesses

